# How gender and religion impact uptake of family planning: results from a qualitative study in Northwestern Tanzania

**DOI:** 10.1186/s12905-019-0802-6

**Published:** 2019-07-22

**Authors:** Radhika Sundararajan, Lauren Mica Yoder, Albert Kihunrwa, Christine Aristide, Samuel E. Kalluvya, David J. Downs, Agrey H. Mwakisole, Jennifer A. Downs

**Affiliations:** 1 Center for Global Health, Weill Cornell Medicine, New York, NY USA; 2 Department of Emergency Medicine, Weill Cornell Medicine, New York, NY USA; 3 Bugando Medical Centre, Mwanza, Tanzania; 4 Catholic University of Health and Allied Sciences, Mwanza, Tanzania; 5 Fuller Theological Seminary, Pasadena, California USA; 6 St. Paul College, Mwanza, Tanzania

**Keywords:** Family planning, Tanzania, Religion, Gender, Qualitative

## Abstract

**Background:**

Women in Tanzania report a high unmet need for both information about and access to family planning. Prior studies have demonstrated the complex and variable relationship between religious faith and beliefs about family planning in sub-Saharan Africa. We hypothesized that a major reason for the poor uptake of family planning in Tanzania is that women and their partners are uncertain about whether pregnancy prevention is compatible with their religious beliefs.

**Methods:**

Twenty-four focus group discussions with 206 participants were conducted in Mwanza, Tanzania between 2016 and 2017: six groups were conducted among Christian men, six among Christian women, six among Muslim men, and six among Muslim women. Among Christians, 98% were Protestants. Focus groups were also divided by gender and religion to facilitate discussion about gender-specific and religion-specific factors influencing family planning utilization. Qualitative data were analyzed using a thematic, phenomenological approach.

**Results:**

We identify two important themes regarding the intersections of religion and family planning practices. First, we report that dynamics of family planning are experienced differently based on gender, and that male authority conflicts with female embodied knowledge, leading to negotiation or covert contraceptive use. Second, religious acceptability of family planning methods is of central importance, though participants differed in their interpretations of their religion’s stance on this question. Most who found family planning incompatible with their faith affirmed their responsibility to give birth to as many children as God would give them. Others found family planning to be acceptable given their moral responsibility to care for and protect their children by limiting the family size.

**Conclusions:**

Both religious tradition and gender dynamics strongly influence the uptake of family planning, with a wide range of interpretations of religious traditions affecting the perceived acceptability of family planning. Regardless of gender or religious affiliation, participants were unified by a desire to live according to religious tradition. Future efforts to improve uptake of family planning are likely to have maximal impact if they are tailored to inform, involve, and empower male heads of households, and to address questions of religious acceptability.

## Background

Women in Tanzania report a high unmet need for both information about and access to family planning. Unmet need for family planning—defined as lack of modern contraception use by a woman who is fecund, sexually active, and does not want a child for at least 2 years—is reported by 61% of women within the first 2 years postpartum in Tanzania [1]. The WHO recommends spacing children by at least 2–3 years to protect maternal and child health [2]. Interpregnancy intervals shorter than 18 months are associated with increases in maternal morbidity and mortality, adverse fetal and infant outcomes, and risk of preterm birth [3]. These benefits have led the United Nations to include universal access to family planning as one of the Sustainable Development Goals, aiming to enable access to modern contraception for an additional 120 million women by 2020 [4].

Family planning information is not uniformly provided to patients seeking healthcare in Tanzania. A recently published cross-sectional study shows that women are less likely to receive family planning information if they have not discussed this topic with their partner [5]. Other studies show that joint decision making about family planning is rare; men are considered primary decision makers regarding household matters, including number of children, and spacing of births [6]. Tanzanian men also report fear that use of family planning will promote promiscuity among their female partners [7]. These factors may explain poor engagement with family planning resources: less than 1/3 of men accompanied their partners to health facilities to receive information about family planning [8]. In the absence of spousal support and lack of information, Tanzanian women have low utilization of family planning methods. Less than 10% of women who sought healthcare over four-month period in rural western Tanzania reported utilizing a family planning method [5]. Further, data demonstrate that women who regularly attend religious services are unlikely to receive family planning information at their health visits [5].

The relationships between religious faith and family planning are complex and variable. While contraception use is prohibited by the Roman Catholic Church, many Protestant traditions permit the use of family planning methods [9]. Christian scripture has been a site of conflicting and contested interpretations of passages in order to support or oppose the use of family planning [10–12]. Diverse perspectives on family planning among Muslims are shaped not only by the Qur’an and prophetic traditions—sources that do not offer unambiguous instructions regarding family planning—but also by political (including post-colonial) contexts in which family planning is often perceived as a ‘colonial and imperial ambition’ of the West [13]. While Qur’anic texts and traditions do not specifically forbid use of contraception, the meaning and relevance of these sources to fertility have also been variably interpreted [14, 15]. Qur’anic texts have been used to support family planning practices by promoting reproductive strategies that mitigate against circumstances that would prevent parents from properly raising their current children. The same texts have been cited to oppose family planning by valuing high fertility and positioning contraceptive use against family interests [16].

Prior studies in sub-Saharan Africa demonstrate that stances on the religious acceptability of family planning vary widely both between religious groups and between individuals within groups, but the desire to access reproductive healthcare is present across groups [17–20]. The vast majority of people in sub-Saharan Africa, and in Tanzania where we work, are deeply religious [21]. To our knowledge, no focus group discussions about family planning and faith among people grouped by the same faith have been conducted. The capacity for religious leaders to influence health behavior was recently confirmed in a large cluster randomized trial, where religious leaders were provided with knowledge and skills to discuss male circumcision in their congregations. This intervention led to a major increase in the uptake of male circumcision [22].

Prior work has described how gender roles impact uptake of family planning, but has not considered how the influences of religion *and* gender intersect. To address this gap, we conducted focus groups among Muslim and Christian men and women in rural northwest Tanzania in order to explore knowledge, religious beliefs, and current practice regarding family planning. We hypothesized that a major reason for the poor uptake of family planning would be that women and their partners are uncertain about whether pregnancy prevention is compatible with their religious beliefs. Our goal was to describe the influences of gender and religious affiliation on utilization of family planning, and consider the potential for interventions within religious communities to promote the uptake of family planning.

## Methods

### Study setting

**Fig. 1:**
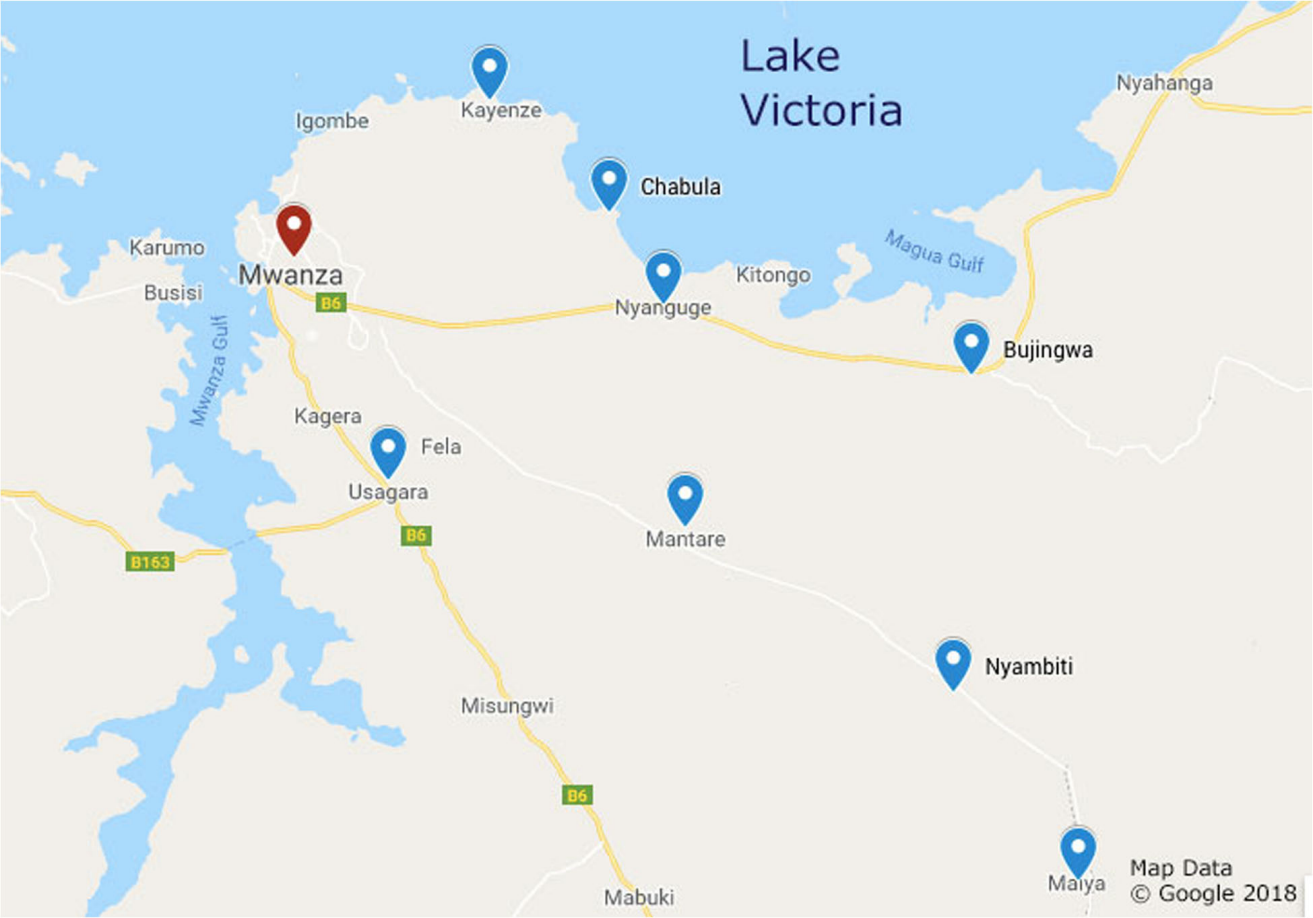
Map of Study Villages. Blue pins indicate location of villages surrounding Mwanza (Red pin) where focus groups were conducted

This study was conducted in rural areas of the Mwanza region in Northwestern Tanzania, near Lake Victoria. Focus group discussions led by facilitators and guided by open-ended questions were conducted in 2016 and 2017. Tanzania has two major religions, Christianity and Islam; we designed our study to include both Christians and Muslims, separated into focus groups by both religion and gender. To streamline the study and minimize costs, our study team traveled together and conducted focus groups in villages that had both a church and a mosque (Fig. 1).

### Data collection

Participants were purposively sampled to ensure that data was collected from a broad range of ages and representatives of the religious denominations in the area, and that participants had knowledge relevant to our topics of interest. We requested leaders from a range of mosques and Protestant churches in each village to invite one to two key informants from among their congregants to participate in our discussions in order to ensure that our participants were active among their respective religious organizations. Women and men were recruited separately to participate, and we conducted focus groups separated by gender in order to allow participants to speak more freely about gender issues and reproductive health issues. This approach was informed by prior research showing that gender roles strongly influence perspectives on family planning, and allowed us to explore areas of agreement or discord among same-gender peer groups. All participants were aged 18 years or older, and provided written informed consent in order to participate. Each discussion was conducted in Kiswahili and led by a team of two native Kiswahili speakers of the same religion and gender as the participants. The leaders guided discussion, which lasted approximately 1 h, using a list of open-ended questions.

Discussions were recorded using a digital audio recorder. The recordings were transcribed verbatim into Kiswahili and then translated into English by a professional translation service in Mwanza. One of the study investigators (A.H.M.), who is fluent in both Kiswahili and English, reviewed both Kiswahili and English transcripts to ensure that original meanings were preserved in the English translations, and to clarify meaning when English translations were unclear. Transcripts were imported and coded using NVivo software V.10 (QSR International, Doncaster, Australia) with the goal of conducting a thematic survey using interpretative phenomenological analysis that would help researchers to understand the attitudes of participants toward a variety of issues relating to family planning [23, 24]. The goal of this method of analysis is to explore participants’ perspectives on an issue, rather than to create an objective description of the issue [24].

In the first step in the analysis, two of the study investigators (L.M.Y., J.A.D.) conducted an independent reading of the transcripts to determine an initial list of major themes. The entire study team, including all focus group leaders, collaborated to refine and clarify themes. This secondary list of themes was then used to code transcripts. As transcripts were extensively re-read, in vivo coding for additional themes by study investigators also occurred. Lastly, illustrative quotations were selected and the text was organized by theme. All study team members reviewed the final text for accuracy and clarity. Ethical permission to conduct this study was obtained from the National Institute for Medical Research in Dar es Salaam, Tanzania and Weill Cornell Medicine in New York, USA.

## Results

A total of 206 people, of whom 106 were Muslims and 100 were Christians, and 104 were women and 102 were men, participated in 24 focus group discussions. The median group size was 9 [interquartile range, 8–9]. Groups lasted a median of 53 [interquartile range, 40–81] minutes. The vast majority of participants reported being married (99%) and having children (91%). Focus group participants were asked to voluntarily provide demographic information, such as age and religious denomination or sect, when they introduced themselves to the group. Among those who volunteered their age (*N* = 66), the median was 40 [interquartile range 30.5–43] years. The primary denominations reported among Christians were the Africa Inland Church, various Pentecostal denominations, Baptists, and Lutherans. Although we did not invite Catholic church leaders to send informants due to Catholic social teaching about contraception, two Roman Catholic parishioners were interested in the group discussions and also attended. Muslim focus group participants did not volunteer their sects when introducing themselves.

Our qualitative data illustrate two important themes regarding the intersections of religion and family planning practices. First, we observe that dynamics of family planning are experienced differently based on gender, and that male household authority conflicted with female embodied knowledge, leading to negotiation or covert contraceptive use. Second, we note that religious acceptability of family planning methods was of central importance, though participants differed in their interpretations of their religion’s stance on this question. Ultimately, the crux of discussions of the acceptability of family planning for both Christians and Muslims came down to the moral responsibility present in both religious traditions to give birth to as many children as God would give them, versus the responsibility to care for and protect children in their families by limiting family size.

### Gender-specific dynamics of family planning

#### Gendered impact of family planning

While the outcome of family planning use is measured at the household level, the impact of the decision to use it or not is borne by women. Our female focus group participants expressed concerns about potential negative repercussions of family planning on their bodies. The discussions revealed that many rumors circulate in communities about side effects of family planning, including gynecological cancer, dangerous alteration of menses, and permanent unwanted sterilization. Study participants also reported a strongly-held belief that women are born with a specific number of eggs, and that women must give birth to all of them or risk being made ill by the eggs that remain in their bodies. This belief, traditional to the Sukuma tribe that inhabits this region, is shared by both Muslims and Christians, and by both genders. Both male and female participants also expressed concern that family planning can produce children with birth defects:

Some believe this family planning has side effects, because if you use the pill you can give birth to a child who has maybe not even fingers. They are not fully developed, maybe they are just half. Maybe the head is twisted sideways, maybe s/he has no legs, maybe handicapped. (Christian man)

Alternatively, family planning was described as central to maintaining maternal and child health. Participants mentioned that failure to space births would prevent their children from getting the attention and care that was crucial to their development. There was general agreement about the practical benefits of limiting the number of children regardless of gender or religion:

We give them a chance of being… [A young child] still needs the mother’s love and you give birth to another child, that means you desert the previous one and you don’t give him/her the same rights equal to the ones of the small child. [By using family planning] we are having ample time to take care of the children we already have with more love and bringing them up better. (Muslim woman)

Another reason which women… follow family planning is the deaths of mother and child… if you look a high percentage of families who do not follow family planning, the mother gets negative effects. First there is exhaustion, exhaustion brings deaths. The mother gives birth one year, she gives birth to a child one year, she hasn’t rested those cells …. She has a child. The following year she has a child. It means it leads even to death. (Christian man)

Beyond protecting the health of women and children, women described additional practical benefits of using family planning, including allowing them time to pursue their own income-generating activities and fulfill their household responsibilities:

Family planning helps me with very many issues because it helps me that I can have a business. Many children in short intervals [means] you cannot do your business. But you plan reproduction, your business goes well, those big children; you give them space and things go very well. (Muslim woman)

Now the man sees the children reach even three, four inside. Now I, the woman, will be unable even to wash clothes, to sweep the house. When he comes in he finds it just dirty. Now it is necessary that he finds a [girlfriend outside of the marriage] because… he’ll find that woman is clean. Maybe she has children, she has only one child. He’ll find the lady there is clean. She washes the clothes. But if he comes to you with five or six children, it just smells of urine inside, so he just has to run away and leave you with this family. (Christian woman)

In contrast to women’s embodied motivations to control their fertility, men commonly described economic factors that drive use of family planning methods. One participant described the stress that accompanies his role as head of household: “It comes like this; you get a family. At times it is too much for you… to give it the important needs. For example, education, a place to sleep, and the needs of food” (Muslim man). Increased household sizes cause stress and strain on men as breadwinners for the family, and motivate interest in family planning:

What causes us men to agree to be advised early to practice family planning is mainly due to the economic situation. If you look at it right now, the economy is already very bad. Men agree easily to family planning because of the life, and low life, you see. I have to reduce the poverty because I am the one to look out for everything, therefore I must be involved so that I bring up few children. (Christian man)

#### Who “decides” on use of family planning?

Our data demonstrate that gender is an important factor with regard to who in the household “decides” whether the couple will utilize family planning. Women described themselves as being motivated to seek family planning because they are personally, physically affected by pregnancies. A Muslim woman stated, “The hardship and problems connected to giving birth are upon the woman. That is why she will make the big decisions herself, according to her health.” Female focus group participants described this *embodied knowledge* justifying why women should have the primary role in deciding on use of family planning.

The one who decides to use family planning is the woman, not the man. Men often don’t agree because he doesn’t have a heavy load. He does not carry the pregnancy. He doesn’t breastfeed. (Christian woman)

Yet, because men are considered heads of households in Mwanza, as throughout much of sub-Saharan Africa, they may be considered the primary decision-makers about family planning for their households:

As I understand family planning, the one with the main decision is the man. He is the one who prescribes for you, ‘Now, my wife, we should use family planning. It is necessary that we go to the clinic, this and that. Indeed, we should use family planning.’ (Christian woman)

For example, we women, it is possible that you… have a man, he does not want you to practice family planning… as for you, that is your husband. You must follow his laws the way he wants. (Muslim woman)

Other participants considered the decision to undertake family planning joint between a husband and wife, where typically the woman introduces the topic. However, the underlying assumption remains that the ultimate authority belongs to the man, and that not all men would listen to their wives’ suggestions:

The one who gets hurt first is the woman. She reaches the conclusion to tell her husband, ‘Sir, my husband, I want to start family planning.’ … Now, if the husband has understanding, the man can receive it directly that it is good. Because the one who gets trouble is the woman. I – the man – don’t get any trouble at the time of giving birth. She is the one who gets the pain, I am just enjoying. (Christian man)

The father gets concerned after being told by the mother about family planning. First, he evaluates himself, the situation he is in in life and economically and… this pace [of rapidly having children] … he sees this idea that his wife brought. If it is useful, then he supports his wife. Therefore, they are both together for the purpose of continuing with family planning. (Christian man)

#### Gendered negotiation and covert contraception use

Our participants explained the conflict between women’s desire to use family planning and the authority of their husbands over household decisions. While women defer to male authority, they practice various strategies of negotiation and persuasion to attempt to gain spousal support for family planning. One Christian woman stated, “We [women] have the ability to confuse the man until he agrees … you convince him until he agrees, so that you join family planning.” In cases where the husband disagrees, some women described employing deception to control their fertility.

You must use extra intelligence – how will you bring this question to him? You will present it. He will find it is now necessary that you use your brain if he is stubborn – because husbands are stubborn. They absolutely don’t agree with this [family planning]. It is necessary that you use an alternative approach, and you see. At what time should I present this question? Which should I use so that I can obtain an agreement more quickly? (Muslim woman)

Clearly the one with the major decision making, it’s the man. That is why at times even women reach the point of using these ways of family planning secretly without informing their men. Some men do absolutely not want to hear something like this, and they have the final say in this matter, which concerns everything regarding marriage. (Christian woman)

Male participants acknowledged that women may utilize family planning covertly, without their knowledge or participation. Women explained that they were taught about family planning during antenatal and postnatal visits to the health clinic, but men did not have a similar opportunity to learn.

We men don’t have that education: the benefit of family planning are these, and the negative impacts are these. That is why the woman, as a woman, she looks at the level of the life which she leads … now it is necessary that the woman uses it [family planning] in secret. (Muslim man)

For some women, the combination of male ignorance about the benefits of family planning and the woman’s own assumptions that her husband would not permit its use justified clandestine use of contraception. Focus group participants described the need for education directed specifically at men with the expectation that such initiatives would empower men to make informed household decisions, and thereby decrease women’s covert use of family planning.

It is necessary that first education must be given so he knows the benefit of family planning. What are the benefits? If he does not use family planning, what are the negative effects? Once he recognizes this, he will be easily involved in implementing family planning. But, if he is not educated, he will not be involved. (Christian man)

### Religion and family planning

#### Religious acceptability of family planning

Our participants expressed a wide range of perspectives regarding the religious acceptability of family planning practices. We found that the lack of direct statements on this topic within scriptural texts creates an area for discussion, leading to varied interpretations as people seek to apply these texts’ teachings to the question of family planning. A number of participants saw family planning as fundamentally opposed by their religious beliefs. Christians who opposed family planning frequently referred to the same scriptural verse (Genesis 1:28) when stating their rationale: “In our religion, we are forbidden to use family planning, because we are told to go and multiply, and fill the land” (Christian woman). A Muslim man similarly argued that family planning practices conflicted with scripture: “The Koran does not allow us because the prophet he says he will be proud on that day when he stands to see the multitude of people is big.” Other participants stated that, while religious texts do not expressly cover this topic, they can infer that family planning would not be supported:

If we go back even to our teachings of the Islamic religion, the lesson of family planning, we don’t have it. Refer even to the teachings of the Prophet Mohammed. This question is not there. Our prophets have given birth to very many children. (Muslim man)

Participants of both religions and genders expressed the belief that using family planning interferes with God’s plan by denying children the right to be born. The Sukuma belief that women are born with a finite number of eggs recurred during these discussions, as participants described a moral imperative to “finish” the eggs given to them by God, while others referred to eggs as unborn children:

There are eggs which you are stopping. It is possible that child would have come to be born and been of help to the community. Now, when you take those medicines, you are going to kill that egg, and you kill God’s plan. (Christian man)

A woman can tell you, ‘We at our home don’t close [use family planning]. I shall give birth until my eggs do what? They finish the ones allocated to me.’ Now you find this woman has a brain. She tells you that, ‘I have already been born with eggs, so I will give them out according to what was given to me by God.’ (Muslim man)

Others saw more ambiguity in whether family planning is acceptable in the context of their religious beliefs. Some described a practical need for family planning that justifies behaviors that may go against perceived religious tradition: “Religion refuses. We should not use family planning… but we go with the times and they have not refused” (Muslim woman). Participants noted that challenges of modern life warrant the use of family planning despite its lack of presence in their religious texts, and suggest that necessity makes family planning, if not approved, at least acceptable.

The Bible is clear, and God has ordered us that we go and give birth, and fill the world. Except, because the way of life has changed for now, the human being enters into the challenge of life. It is better to at least use family planning because life is difficult. (Christian man)

There are diseases the woman may have, like diabetes, high blood pressure. These are among all the things that can cause her to use family planning. Even though in our religion of Islam, these things are not there; in our faith there is no family planning. It is not there at all, but according to the situation right now, it has to be done. (Muslim man)

Many others voiced an understanding of their religious beliefs that is consistent with, and even encouraging of, the use of family planning. These interpretations focused on moral lessons derived from religious texts, such as caring for children and living within one’s means, and concluded that family planning use was consistent with these moral standards.

When God says that you should fill the earth, he did not mean we should just give birth, that we should just give birth haphazardly. He meant that you give birth to those children you should be able to feed them with your income … You should give birth to the children that you can take care of in your life … If you do not take care of them, you just leave them? That is also a sin. (Christian woman)

God has started this question. He has seen it – yes indeed – because he said first when you want to marry, you marry in accordance with your position. God has admonished us, first even if you want to marry, marry the women you are able to keep. But, you must fulfill their needs. There we came to look, and in this question of children, the religion is also involved to tell us we should give birth in relation to our capacity. It has admonished us we should give birth in accordance to our economic capacity, the way we are. Yes, indeed that is how religion tries to be involved. (Muslim man)

#### Could family planning education be provided by religious leaders?

Most participants described themselves as desiring information on these topics, but their opinions on the possibility of receiving this information from a religious source varied. When asked how they would feel if their religious leader discussed family planning, some participants of both religions stated that family planning discussions were not appropriate in that setting, saying, “Questions of family planning? Those are completely different from the topic of religion” (Muslim man). This led some participants to push back against the suggestion that family planning education could take place in a religious setting:

You see, [the Imam] will have opened our ears … so that we get the exemplification that family planning is ok. But on the other side, some people will contradict him, because he is not allowed to tell us in the mosque. (Muslim woman)

If my leaders tell me about family planning, I wouldn’t feel good because the word of God says ‘Go into the world. Fill this country, and what belongs to Caesar leave it to Caesar.’ Therefore, it will be good to get this education on the side of Caesar. (Christian man)

In contrast, others were enthusiastic about the idea of having their religious leaders speak on this topic, attributing this to the trust they have in their religious leaders to provide guidance on a difficult topic, and the inherent authority of information from such a source:

It would be a good thing [if religious leaders provided family planning education] because indeed… it will be easy. Men for men. Therefore if they [male religious leaders] deliver it [family planning education] it will indeed be better because we ourselves [women] we are taught and then the man is at home. The woman cannot teach the man at home, and some men don’t listen at all. (Muslim woman).

If… in church they talk about this matter… you must feel good because they give quality education to the believers and the whole society, not only in church. It means once you are taught in church straight away even outside you will provide it, you will teach others. (Christian man)

## Discussion

Our study illustrates the major dual influences of gender and religion on the dynamics of family planning in a region of rural sub-Saharan Africa, and contributes to a growing body of knowledge demonstrating how these factors shape engagement with family planning resources [18, 19, 25]. Our findings are congruent with other studies that have described how gendered roles lead to conflict between partners because the domain of family planning is regarded as “a woman’s matter”, though men are still considered decision makers at the household level (including use of family planning) [6, 26, 27]. Our data illustrate that men are considered to have the *authority* in the household to decide whether family planning is acceptable. Women’s interest in undertaking family planning is driven by knowledge of the physical costs of pregnancy and unspaced births, and desire to preserve the health of themselves and their children; their embodied *knowledge* informs their perspectives on fertility and childbearing.

Both men and women describe a disparity between male authority and female embodied knowledge as a cause of conflict and a complicating factor in the decision to use family planning. Male authority is challenged in some cases to serve a pragmatic outcome, which leads to men’s authority not uncommonly being subverted. Female participants describe processes of manipulating spouses to gain ideological support for family planning; in absence of support, many utilize contraception covertly. Such covert contraception use is common in sub-Saharan Africa [28]. Our participants described an ideal scenario as one in which both spouses are involved in the decision-making process, but cited lack of male understanding about family planning as a major barrier to such joint decision-making. Our work supports initiatives to provide family planning education to empower men, which could increase household joint decision making [29], decrease the need for women’s covert use [30], and increase uptake of family planning in areas with high unmet needs [31]. Both men and women in our study agreed that this type of health education would benefit their households.

A unique contribution of our work is that it provides insights into the potential that community-based education holds to increase the uptake of family planning. Tailoring education about family planning so that it is provided to men, who often lack other sources of information about this topic, and so that it incorporates discussion of religious beliefs, appears to be of critical importance. We observe a striking point of unity among men and women of both faiths: the desire to live in accordance with religious teachings. We did not appreciate a difference between Christian and Muslim perceptions or use of family planning, but within both faiths noted a diversity in perspectives about how religious teachings integrate with family planning. While some participants were staunchly opposed to use of family planning, many sought coherence between experiential, practical knowledge about family planning and faithfulness to the teachings of their scriptures. For example, our participants emphasized the relevance of modern life stressors in interpreting scripture, or they considered family planning as acceptable because it is not specifically discussed in religious texts. Others acknowledged confusion regarding questions about family planning, because this topic is not explicitly addressed in scripture, and looked to apply the larger moral lessons of their religious tradition to specific questions of modern life. Many participants reached the conclusion that family planning is a moral good, and is justified by their religious beliefs. Our results reveal practical topics that need to be addressed in order to provide effective family planning educational initiatives within communities, such as northwestern Tanzania, where religious beliefs strongly influence health behaviors.

Our data suggest that uptake of family planning could be increased by providing education about family planning in the context of religious interpretation. Tanzania, like other countries in East Africa, is deeply religious: only 1.6% of its population reports no religious affiliation, and 93% rate faith as “very important” to them [21]. Other research has shown that women who regularly attend religious services are less likely to receive information on family planning from healthcare facilities [5]. We previously showed that church attenders in Tanzania are eager to learn about and discuss reproductive health interventions in the context of their religious beliefs [32] and that the uptake of male circumcision could be increased when church leaders received education about this health topic, which they then imparted to their congregations [22]. This work demonstrates the strong influence that religious leaders have in communities, and the potential to impact other healthy behaviors by working within religious communities. Our work and a cross-sectional survey from Malawi [20] support the potential of religious leaders to impact the health behavior of the whole community, making them potentially valuable allies in promoting health interventions.

Our study has a few limitations. First, given the nature of the discussion topics, social desirability bias may have influenced some participants to under-report certain behaviors or beliefs, and therefore constrain our understanding of factors relevant to family planning. Second, a limitation of the analysis is that it only reflects perspectives of community members. Multiple perspectives on engagement with family planning would produce a more comprehensive picture of factors at play within this community, including representation from religious leaders and family planning clinic staff, for example.

## Conclusion

Both religious tradition and gender dynamics strongly influence the uptake of family planning. Our data illustrate a wide range of interpretations of religious tradition regarding family planning, all of which were unified by a desire by participants to live according to religious tradition. While for some, this led to the absolute conclusion that family planning opposed their religious faith, many applied their interpretations of religious morality to the question of family planning and concluded that it was not only acceptable, but fully supported under their religious moral framework. Future efforts to improve uptake of family planning are likely to have maximal impact if they are tailored to inform and empower male heads of households, and directly address questions of religious acceptability.

## Data Availability

The datasets used and analyzed during the current study are available from the corresponding author on reasonable request.
